# Immune pathogenesis of idiopathic granulomatous mastitis: from etiology toward therapeutic approaches

**DOI:** 10.3389/fimmu.2024.1295759

**Published:** 2024-03-11

**Authors:** Xiaoli Wang, Xiujing He, Junzhi Liu, Haiyan Zhang, Hangyu Wan, Jing Luo, Jiqiao Yang

**Affiliations:** ^1^ Breast Center, Department of General Surgery, West China Hospital, Sichuan University, Chengdu, China; ^2^ Clinical Research Center for Breast, State Key Laboratory of Biotherapy, West China Hospital, Sichuan University, Chengdu, China; ^3^ Laboratory of Tumor Targeted and Immune Therapy, State Key Laboratory of Biotherapy, West China Hospital, Sichuan University, Chengdu, China; ^4^ West China School of Medicine/West China Hospital, Sichuan University, Chengdu, China; ^5^ Department of Breast Surgery, Sichuan Provincial Maternity and Child Health Care Hospital, Chengdu, China

**Keywords:** idiopathic granulomatous mastitis, autoimmune, autoimmunity, etiology, pathogenesis

## Abstract

Idiopathic granulomatous mastitis (IGM) is a noncancerous, chronic inflammatory disorder of breast with unknown causes, posing significant challenges to the quality of life due to its high refractoriness and local aggressiveness. The typical symptoms of this disease involve skin redness, a firm and tender breast mass and mastalgia; others may include swelling, fistula, abscess (often without fever), nipple retraction, and peau d’orange appearance. IGM often mimics breast abscesses or malignancies, particularly inflammatory breast cancer, and is characterized by absent standardized treatment options, inconsistent patient response and unknown mechanism. Definite diagnosis of this disease relies on core needle biopsy and histopathological examination. The prevailing etiological theory suggests that IGM is an autoimmune disease, as some patients respond well to steroid treatment. Additionally, the presence of concurrent erythema nodosum or other autoimmune conditions supports the autoimmune nature of the disease. Based on current knowledge, this review aims to elucidate the autoimmune-favored features of IGM and explore its potential etiologies. Furthermore, we discuss the immune-mediated pathogenesis of IGM using existing research and propose immunotherapeutic strategies for managing this condition.

## Introduction

Idiopathic granulomatous mastitis (IGM), first described in 1972 (1), is a nonspecific, refractory inflammatory breast disease mostly affecting young parous women (2, 3). It is characterized by the sudden onset of breast lumps, abscesses, sinus tracts, and ulcers (2). IGM was once considered an orphan disease; however, in recent years, the incidence has been calculated to compromise 1.8% of benign breast diseases (4) and 24% of inflammatory breast diseases (5). Interestingly, the prevalence of IGM varies regionally or racially, with higher rates observed in Middle Eastern, Mediterranean, Asian, and Hispanic populations compared to Western Caucasian populations (6–9).

IGM is not cancerous, but can be highly recalcitrant and locally aggressive, significantly impacting patients’ quality of life and potentially leaving permanent cosmetic deformities (10). As a mysterious entity to date, IGM is still difficult to diagnose and treat. The etiology remains largely unclear and clinicians have an inconsistent understanding of the disease, resulting in a lack of a gold standard for IGM management. While a portion of patients respond to corticosteroids, antibiotics, doxycycline, and methotrexate (2), relapse rates have been reported as high as 5-78% (5, 11). Surgery is not a common treatment, but some patients underwent multiple surgeries and, in extreme cases, mastectomies due to refractory pain, abscesses, fistulas, sinuses, and healing complications (12–15).

Most commonly, IGM is characterized by a firm, tender breast mass, skin redness, and mastalgia (3). Other symptoms include swelling, fistula, abscess (often without fever (16)), nipple retraction, and peau d’orange appearance (17). The extramammary manifestations includes erythema nodosum and regional lymphadenopathy, present in a quarter of patients (18). Ultrasound, as an imaging modality, is commonly used for diagnosis of IGM, indicating irregular hypoechoic masses, multiple hypoechoic masses, parenchymal heterogeneity, or heterogeneous echogenic areas on ultrasonographic examination (19). The common mammographic features are focal or diffuse asymmetric density (20), while magnetic resonance imaging is not routinely utilized in the evaluation of IGM (9).

Clinically and radiologically, IGM often mimics breast abscess or malignancy, especially inflammatory breast cancer (14), therefore definitive diagnosis relies on core needle biopsy and histopathologic examination. IGM is histologically characterized by lobular-centered epithelioid noncaseating granulomas, with occasional sterile microabscesses. Epithelioid histiocytes are predominantly neutrophils and eosinophils. Langhans cells, lymphocytes, and plasma cells generated by the eosinophilic cells- dominated inflammatory reactions are also observed in the lobular center of the breast parenchyma. Depending on the disease severity, the inflammatory responses may extend and obliterate the lobular-centric pattern. It should be noted that the diagnosis of IGM is not clear until inflammation caused by other specific causes has been excluded, including inflammatory breast malignancies, tuberculosis, infective mastitis caused by known pathogens, sarcoidosis, Wegener’s granulomatosis, granulomatosis with polyangiitis, diabetic mastopathy, foreign body reaction, and IgG4-related sclerosing mastitis (3, 10, 21).

**Figure 1:**
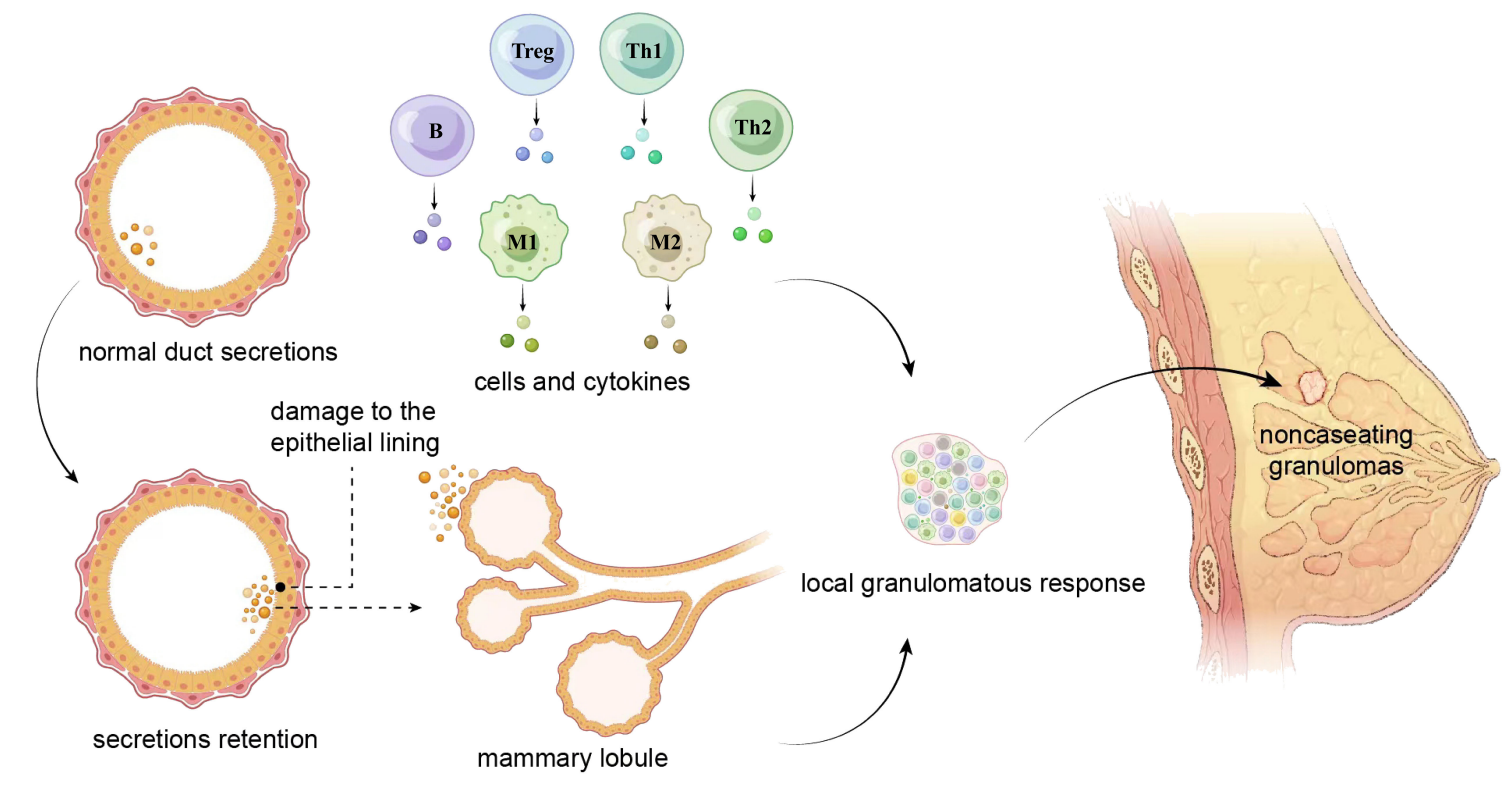
A tentative pathogenic pathway for IGM proposed by Benson and colleagues. The damage of the epithelial lining caused by retention of ductal secretions leads to the leakage of content from the lumen into the surrounding lobular connective tissue, and the extravasation initiates local inflammatory reactions in which lymphocytes and macrophages are involved. These cells migrate to the periductal area, produce cytokines, and trigger a local granulomatous response that pathologically presents with noncaseating granulomas. Th, helper T cell; Treg, regulatory T cell; M, macrophage; B, B lymphocyte.

Currently, the most widely supported etiological theory suggests that IGM is an autoimmune disease (22). The fact that some patients responded well to steroid treatment and the observation of concomitant erythema nodosum (23) or other autoimmune conditions further supported the autoimmune nature of the disease. Nonetheless, previous studies have failed to find an association between antinuclear antibody (ANA) levels and the risk, medical response, or recurrence of IGM (24). Benson and colleagues proposed a tentative pathogenic pathway for IGM: 1) the ductal epithelial lining damage that is possibly secondary to the retention of ductal secretions; 2) efflux of ductal contents from the lumen into the surrounding lobular connective tissue; 3) development of local inflammation in response to extravasated ductal contents; 4) migration of lymphocytes and macrophages into periductal zones; and 5) local granulomatous response with the formation of noncaseating granulomas (**Figure 1**) (25). Other possible etiologies include hyperprolactinemia, hormonal imbalance, microbiological agents, and genetic factors (13, 26). In this manuscript, we provide critical insights into the current understanding involving the pathogenetic features of IGM, the complex natures and potential therapeutic strategies of a condition that is confounding the medical community.

## Autoimmune-favored features of IGM

### Erythema nodosum and arthritis

Erythema nodosum (EN), first described in 1987, is a rare but typical systemic manifestation of IGM (27). EN is blood vessel-associated hypodermal septal inflammatory panniculitis characterized by reddish, tender nodules, typically of bilateral subcutaneous pretibial regions and occasionally seen on arms (28). The reported frequency of EN among IGM patients ranges from 6.6% to 58.1% (28–31). The results of a retrospective, comparative parallel-arm study involving 43 patients with IGM coexisting with EN and 43 with IGM only suggested that the coexistence with EN presented higher fistula distribution and recurrence rates, indicating a worse prognosis for IGM patients (28, 32). In addition, IGM patients with EN often have more extensive lesions or bilateral breasts involved, suggesting a more severe disease (29). Inflammatory arthritis/arthralgia is another extramammary manifestation of IGM with a lower incidence than EN (33). In clinical observations, regression of arthritis in IGM patients is usually synchronized with the remission of local (mammary) symptoms (34). Despite a low occurrence, the simultaneous involvement of EN and arthritis in IGM patients has been reported previously (35). Therefore, EN and arthritis might have autoimmune mechanisms similar to those of IGM in disease development.

EN can be triggered by various antigenic stimuli (34) and is associated with a range of pathological conditions, such as drug reactions, infection, tuberculosis, and malignancies (36). Similar to IGM, EN also has an unelucidated pathogenesis. It has been hypothesized that increased immune complexes, cytokines, and chemokine levels in the blood might lead to EN in severe IGM cases by triggering septal panniculitis induced by neutrophilic recruitment and activation in pretibial subcutaneous tissue (29). It is widely believed that systematic involvement, such as EN and arthritis, supports an autoimmune component in etiology (34, 37).

### Coexistence with other autoimmune diseases

Apart from systematic symptoms, IGM has also been reported to coexist with autoimmune diseases, such as systemic lupus erythematosus (SLE) (38) and Sjögren’s syndrome (39, 40), simultaneously within an individual. This simultaneous presence of two or more autoimmune diseases in a patient is defined as polyautoimmunity and is considered a common phenomenon in rheumatic and autoimmune diseases (41). As reported, 33% of patients suffering from SLE and 1/3 of patients with Sjögren’s syndrome have developed another autoimmune disease (42, 43). The polyautoimmunity among IGM patients indicates the importance of shared etiopathogenesis with other autoimmune diseases.

### Seasonal fluctuation of disease onset

Although there is no solid statistical evidence, seasonal fluctuations are noticed in the onset of IGM (32, 44). In a retrospective study of 44 patients treated for IGM, an increased incidence of IGM was observed during the warm seasons from 1994 to 2018 (44). A similar pattern was noticed in a single-institutional retrospective analysis of 134 IGM patients, with a slightly higher incidence observed in summer and late spring (32). However, despite the observation of the above trends in the distribution of onset times, the above two studies were unable to draw statistical conclusions. The small sample size may account for this, especially when divided into twelve months.

Seasonality has been well recognized in autoimmune disorders (45). For example, it has been suggested that the onset of autoimmune encephalitis was dominant in summer-autumn (46). Moreover, rheumatoid arthritis (RA) activity in the upper and lower extremities peaks in spring (47). The loss of tolerance to self-antigens contributes to the development of autoimmune diseases (48). Environmental factors with seasonal fluctuation patterns, such as temperature and humidity, vitamin D levels that varied with ultraviolet radiation exposure, and melatonin secretion were indicated to influence disease onset and progression (45). Vitamin D levels are associated with disease activity in multiple sclerosis (MS), SLE, psoriasis, and RA. Melatonin exerts anti-inflammatory properties by affecting different arms of the immune system including but not limited to the inhibition of inflammatory cytokines (49). Melatonin levels, which reach their minimum in spring, may aggravate MS and SLE (45). The incidence increases in spring and early summer in children with primary immune thrombocytopenia (ITP) (50). A similar pattern has been observed in acute adult ITP, which may be attributed to seasonal variations in certain areas of infection that lead to proinflammatory cytokine release and human antigen mimicry (32). In addition, seasonal variations have also been reported in the production of hormones (e.g. growth hormone and insulin-like growth factor) and self-reactive autoantibodies (e.g. anti-Jo-1 autoantibodies, anti-SRP autoantibodies, anti-proteinase 3 antibodies, and autoantibodies to islet cell antigens or pancreatic tyrosine phosphatase) (51). In IGM patients, although the current evidence of seasonal fluctuations and hidden mechanisms is far from solid, the trend observed adds to the suspicion of its nature as an autoimmune disease.

## Triggering events and precipitating factors of IGM

### Pregnancy and parturition

IGM is typically found in childbearing-age women with a history of pregnancy and/or lactation within 5 years (52–55). In a multicenter retrospective analysis including 22 breast centers with a total of 720 IGM patients, the overall incidence rate of the first recurrence was 17% (122/720), and the re-recurrence after treatment was 3% (22/720), with a statistically significant association between recurrence and the history of pregnancy (p=0.0008) and of breastfeeding (p=0.03), respectively (5). Therefore, the history of pregnancy and/or lactation (rather than the moment of pregnancy and breastfeeding) is seen as a risk factor for IGM recurrence.

The relevance between history of pregnancy and/or lactation and autoimmune diseases has been discussed from time to time. Both local and systemic factors accounted for the pathogenic mechanisms of IGM. Benson and his colleagues proposed a tentative pathogenic pathway for IGM centered on the mammary ducts. Possibly secondary to the retained ductal secretions, damage to the ductal epithelial lining induced the outflow of contents from the lumen into the surrounding lobular connective tissue which in turn developed a local inflammation in response to extravasated contents. Macrophages and lymphocytes involved in the above mentioned local inflammation migrate to the periductal zones and induce a local granulomatous response characterized by the formation of noncaseating granulomas (25). Additionally, damage to the ductal epithelium may occur as a result of milk stasis in the absence of breastfeeding (33). Systemic factors are prominent among the effects of pregnancy on many autoimmune diseases (25). RA often began several months after delivery, the disease activity flared in 46.7% of patients postpartum but interestingly improved in 60% during pregnancy, as reported in a meta-analysis (56). Another study also observed an increased risk of new-onset RA during the first 3 months postpartum, especially in primigravida for whom this risk increased to almost 11-fold (57). Up to 30% of young women with Graves’ disease had a history of pregnancy in the 12 months prior to the onset (58). It is indicated that pregnancy is a significant risk factor for susceptible women and postpartum autoimmune diseases are not rare. There is a great chance that the onset and progression of autoimmune and/or inflammatory diseases are regulated by T cell cytokine-mediated responses not only during the gestational and the postpartum periods but also decades after pregnancy, with progesterone and estrogens inducing a T helper 2 (Th2)-dominated response which further prompts the worsening of Th2-type autoimmune diseases, and impressing the Th1/Th17-dominated response which facilitates the improvement of Th1/Th17-type autoimmune diseases (59). Therefore, the pathogenic mechanism and the exact correlation between IGM and a recent history of pregnancy and/or lactation need to be further illuminated.

### Hormonal disorders

Hormonal imbalance has a notable impact on the pathogenesis of IGM (60–62). Diagnosed with hyperprolactinemia secondary to a prolactinoma, IGM patients in two case reports improved after treatment with prolactin inhibitors (63, 64). In nonpregnant IGM women with schizophrenia or bipolar disorder, hyperprolactinemia was secondary to risperidone therapy (65–67); however, in some cases, prolactin (PRL) levels may be normal (68).

The relationship between PRL and immune system has been elucidated to a great extent in the last decade, shedding new lights on the field of immunoendocrinology. Hyperprolactinemia has been described in the active phase of some non-organ-specific autoimmune diseases (e.g. SLE, RA) and organ-specific autoimmune diseases (e.g. celiac disease, type 1 diabetes mellitus, Addison’s disease, and autoimmune thyroid disease) (69). A study recruiting 102 patients with hyperprolactinemia showed a possible autoimmune condition in which levels of interleukin-4 (IL-4) and thyroid peroxidase antibodies (TPOAb) are higher (70). PRL increased the Th1 lymphocytes-induced synthesis of interferon gamma (IFN-γ) and IL-2, and activated Th2 lymphocytes-induced production of autoantibody, therefore of worthy interest was the association between hyperprolactinemia and levels of cytokines and specific/nonspecific antibodies in autoimmune diseases (69). An animal experiment concluded that persistently elevated levels of serum prolactin interfered with B cell tolerance induction by impairing B cell receptor (BCR)-mediated clonal deletion, deregulating receptor editing, and lowering the threshold for anergic B cell activation, thereby promoting autoreactivity (71). Different from the sex hormones that experience a cliff-like drop at delivery and a slow recovery postpartum, prolactin increases slightly during pregnancy and rises exponentially at delivery and during lactation (72). Future studies may confirm the specific association between IGM and hormonal disorders and unravel the pathogenic mechanism.

### Trauma and duct epithelial damage

Trauma and ductal perforation have been reported as triggering factors for IGM. The proposed mechanism of IGM involves the following steps: ductal epithelial damage, leakage of luminal secretions into surrounding lobular connective tissue, local inflammation, migration of macrophages and lymphocytes into the region, and local granulomatous inflammatory response (17). Secretion extravasation, as a result of ductal damage, causes migration of macrophages and leukocytes into the tissue, leading to local inflammation (33). In contrast, another tentative pathogenesis of IGM advocates that an insult to the ductal epithelial cells, perhaps by directing against antigens within lactations, triggers the formation of local granulomatous (36). Specifically, ductal perforation elicits an inflammatory response, and the subsequent immune presentation of mammary antigens induces autoimmunity, contributing to reactive T cell-mediated inflammation and centrilobular granuloma formation (9). There is a strong possibility that skin lesions may be a consequence of epitope spreading following an immunological event activated within the breast tissue (36). In addition to the lactation process, trauma may be responsible for ductal epithelial damage. Other trauma-related conditions suspected of causing epithelial damage have been reported, such as one case of underlying IGM was exacerbated by ductoscopy. A 41-year-old woman in this case report went to the outpatient clinic with a 2-year history of right-sided pathologic nipple discharge, excluding remarkable medical history and drug history. She developed pain and a 4-5 cm palpable mass in the lower outer quadrant four days after ductoscopy. The patient initially responded well to antibiotics but ten days later presented with abscesses that were finally incised and drained by surgery. A few days after a week of slow recovery, a new red palpable mass developed in the upper outer quadrant of the breast, and the histopathology of an ultrasound-guided biopsy showed granulomatous mastitis (73). Furthermore, trauma and ductal perforation are emerging as risk factors for other systemic immune disorders. Twenty nine patients with traumatic brain injury completed a follow-up of 3 years and the result suggested significantly worse pituitary dysfunction in antipituitary antibody (APA)-positive patients than in APA-negative patients (46.2% *vs* 12.5%, P=0.04), with a corresponding OR of 2.25 (95% CI 1.1–4.6) (74). A detailed, responsible, and practical investigation of the trauma mechanism may be a high priority for further research in some patients with IGM.

### Smoking

A recognition is led to that smoking is possibly one of the risk factors for IGM, but no definite conclusions have been drawn thus far. The reported proportions of IGM patients who smoke were 0%-77.8% (55, 75–78). A 11-year retrospective study of 77 patients with IGM found two groups (high flare *vs* low flare) were largely comparable except for smoking as a risk factor. Specifically, smokers added to 10 times greater odds of having a high IGM flare than non-smokers (P = 0.031) (12). A multicenter and retrospective analysis of 720 patients with IGM also found a significant association between smoking and IGM recurrence (p=0.01) (5). Therefore, investigators consider smoking as one of the risk factors for IGM recurrence.

The association between smoking and autoimmunity has long been proposed (79, 80). Epidemiologic studies have stated that tobacco smoking is associated with the development, disease course, symptom severity, and outcome of autoimmune diseases, such as RA, systemic sclerosis (SSc) and SLE (81). Smoking modulates immune system through multiple and complex mechanisms, including the immunosuppression, induction of apoptosis, induction of the inflammatory response, alteration of cytokine balance and the synthesis of anti-DNA antibody generated by DNA damage (81). It promotes autoimmune diseases by releasing intracellular proteins from reactive oxygen species (ROS)-activated or injured cells, altering antigen presentation in cigarette smoke-impaired cells, modifying regulatory B- and T-cell functions, dysregulating DNA demethylation and upregulating immune genes (12, 82–84). The existence of susceptible genes further expands the understanding about the role of smoking in autoimmunity (81). However, a clearer relationship between IGM and smoking-caused pathogenic mechanism still remains to be elucidated.

### Microorganisms

IGM presents as an inflammatory mass in the peripheral tissues of the breast or simultaneous peripheral (rarely central) inflammation at multiple sites with abscesses and/or inflammation and ulceration of the skin (55, 85–87). Partly for this reason, infections or microorganisms are naturally considered as a potential risk factor for IGM. A fine needle aspiration culture of the abscess in a female patient with IGM revealed *Enterococcus* avium (88). Tissue culture from a repeat biopsy in a 28-year-old woman grew *Mycobacterium mucogenicum*, a rare cause of skin and soft tissue infections (89). As a possible infectious etiology, *Corynebacterium* species (90), especially *Corynebacterium kroppenstedtii (* 91, 92 *)*, have been identified in the progression of IGM. Microorganisms detected by culture in previous studies are cluttered, random, and lack regularity, making it difficult to guide clinical practice.

Infections provide necessary antigens by mimicking or altering self-antigens, leading to an increase of overall immune reactivity level or an antigen “spillage” (93, 94). In addition, infections serve as inflammatory context that favors the activation of innate immune responses (95). Importantly, the Epstein–Barr virus has been implicated as an inciting agent for several autoimmune diseases, including MS and SLE (96, 97).

In the apparent absence of infection, microorganisms also influence the initiation and progression of an autoimmune responses. Populations of commensal microorganisms (“microbiota”) profoundly affect the induction of diseases such as inflammatory bowel disease on humans (98). Changes in the composition of the microbiota are common in autoimmune diseases, giving rise to a condition termed dysbiosis (99, 100). For example, Enterococcus gallinarum, found in the liver of SLE patients with cross-reactivity, developed an autoimmune response to the Ro antigen (101).

### Others

The primary function of Alpha-1-antitrypsin (AAT), a glycoprotein synthesized by hepatic cells, is to prevent the destructive effects of proteases secreted by activated neutrophils. Schelfout et al (102) demonstrated AAT deficiency in a 37-year-old female patient with IGM. According to current studies, we authors define no immunological events, but hypothesize that AAT deficiency could be one of the etiologic factors; therefore, further investigations are needed to support this argument.

## Immune pathogenesis of IGM

### Cellular dysregulation

**Figure 2:**
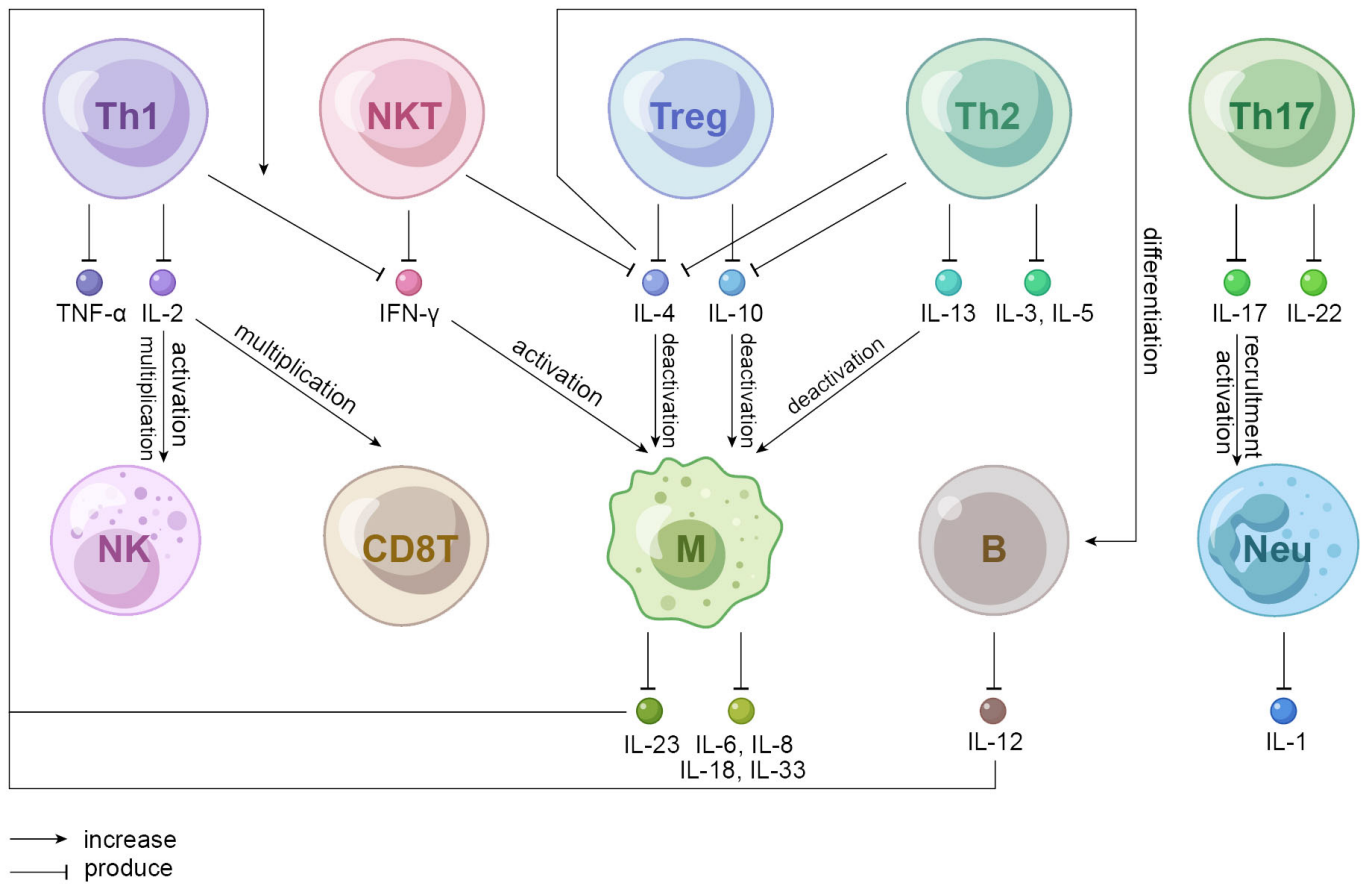
Mechanistic hypothesis of autoimmune cells involved in IGM. Th1 cells produce IL-2 that activates NK cells and multiplicates CD8T and NK cells. IFN-ɣ produced by NKT and Th1 cells activates macrophages that produces a variety of interleukins among which IL-23 increases Th1-produced IFN-ɣ. IL-4 (produced by NKT, Treg, and Th2 cells), IL-10 (produced by Treg and Th2 cells) and IL-13 (produced by Th2 cells) all deactivate macrophages; IL-4 also induces the differentiation of B cells. Th17 cells produce IL-22 that promotes proliferation, remodeling, and repair of tissues and organs to maintain innate host defense mechanism and IL-17 that participates in the activation and recruitment of neutrophils. Th, helper T cell; Treg, regulatory T cell; NKT, natural killer T cell; NK, natural killer cell; CD8T, cluster differentiation 8 positive T cell, also known as CTL, cytotoxic T lymphocyte; M, macrophage; B, B lymphocyte; Neu, neutrophil.

Hypothesis that autoimmunity plays a role in IGM has long been considered, and the dominance of T lymphocytes found in some studies partially forms the basis for this theory (**Figure 2**). A study recruiting 51 pathologically proven IGM patients (26 in activity; 25 in remission) and 28 healthy volunteers as controls pointed that lymphocyte subsets in the peripheral blood determined their respective impacts on etiopathogenesis. To be more specific, the percentage of Th cells was lower in patients with IGM than controls regardless of disease severity, perhaps because the recruitment of Th cells to granulomatous breast tissue contributes to the low T cell counts in the peripheral blood of patients. However, the absolute count of cytotoxic T lymphocytes increased as Th cells decreased, presenting with higher percentage and greater absolute count both of natural killer (NK) cells and natural killer T (NKT) cells in IGM patients than controls (8). NKT cells suppressed granuloma formation by modulating the production of IFN-ɣ and IL-10 according to research performed on mouse livers (103). That is, observed changes in T cells, NK cells and NKT cells imply systemic immune dysregulation in patients with IGM which is recognized as a localized form of granulomatous disorder. In a large cohort study of granulomatous mastitis in which microbiologic studies were negative, IGM showed significantly more plasma cells than tuberculous mastitis in terms of histologic features. Current understanding possibly accounts for the autoimmune aetiology, which would draw in humoral responses and thus more plasma cells (104). A recent study suggested that CD68- and CD163-positive M2-type macrophages were privileged in the course of plasma cell mastitis (PCM) and granulomatous lobular mastitis (GLM). CD 57-positive NK cells even provided a practical basis for clinical staging of GLM. The Neutrophil-to-lymphocyte ratio (NLR), one of the preoperative inflammatory parameters, was significantly associated with IGM recurrence, highlighting that a high NLR predicted poor patient outcomes (105, 106).

### IL family/cytokine

Cytokines regulate the growth, differentiation, maturation, and response of cells (**Table 1**). In general, levels of IL-4, IL-5, and IL-13 increased in allergic diseases; levels of IL-17 and IL-23 increased in autoimmune diseases; and levels of IL2 and IFN-ɣ increased in sarcoidosis and granulomatous diseases (112). The levels of IL-33 (107), IL-6 (108), IL-8, IL-10, IL-17 (109), IL-22, and IL-23 (110) were all significantly higher in IGM patients than healthy controls. IL-6 level was even significantly higher in patients with severe disease than those with mild and moderate disease, indicating that IL-6 is a biomarker for assessing disease severity in IGM (108). However, a recent study showed that values of IL-1β, IL-10, and IL-18 were significantly lower in the acute IGM group than the control group (111).

**Table 1:** Reported cytokines associated with IGM.

IL family	Type of research	Sample size	Method	Results (statistically significant)	Meaning	Reason
IL-33 [ (107)]	Case–control study	*25 IGM patients* *32 BC patients* *30 HCs*	Human IL-33 (Platinum ELISA kit, BMS2048/BMS2048TEN, eBioscience, Vienna, Austria)	IGM>BC>HCs	Distinguishing diagnoses of IGM and BC (along with the radiological and pathological findings).	The induction of T-helper type 2 immune response
IL-6 [ (108)]	Case–control study	*11 IGM patients* -mild (n = 1) -moderate (n = 4)-severe (n = 6) *7 HCs*	ProcartaPlex magnetic bead-based multiplex assay (Cat. No. EPX180-12165-901; eBioscience, Vienna, Austria)	1. IGM >HCs 2. IGM: severe>moderate 3. positive correlation with time to resolution	Biomarkers for disease severity and time to resolution in IGM patients.	The stimulation of IL-1 and Toll-like receptors
IL-8 IL-10 IL-17 [ (109)]	Case–control study	*47 IGM patients* -active (n=21) -remission (n=26) *30 HCs*	Human enzyme-linked immunosorbent assay kits (Elabscience^®^, Houston, USA).	IL-8、IL-10、IL-17: IGM>HCs IL-10:active IGM>HCs	Indicating the presence of serious immune dysregulation in IGM patients.	IL-8 mRNA is upregulated by the deficiency of NADPH oxidase in CGD
IL-22 IL-23 [ (110)]	Case–control study	*26 IGM patients* *15 HCs*	Elabscience Human IL-22 ELISA Kit, cat. no. E-EL-H0107; and Elabscience Human IL-23 ELISA Kit, cat. no. E-EL-H0106	IGM >HCs	Supporting the etiopathogenesis of IGM in favor of the autoinflammatory thesis.	Activated CD4+ cells initiate inflammation by releasing inflammatory cytokines
IL-1β TNF-α IL-10 IL-18 [ (111)]	Case–control study	*32 IGM patients* -active (n=19) -remission (n=13) *18 HCs*	Flow cytometry (FACSLyric™,BD, USA)	IGM<HCs active IGM<HCs	Cytokines, which play roles in known autoimmune and Granulomatous reactions, especially cytokines related to Th1 and Th17, might not play a role in the etiopathogenesis in GM patients.	-Inflammasome Th1 may not play roles in IGM etiopathogenesis。 -The cytokine measurement method was bead-based immunoassay on Flow Cytometry instead of ELISA.

BC, breast cancer; HCs, healthy controls; ELISA, enzyme-linked immunosorbent assay; NADPH, nicotinamide adenine dinucleotide phosphate; CGD, chronic granulomatous disease.

### HLA

A study covering 48 patients with IGM and 50 healthy controls reported that the frequency of human leukocyte antigens (HLA)-A*10, HLA-A*2403, HLA-B*18 and HLA-DR*17 was significantly higher in the patient group than the control group (p=0.012, p=0.012, p=0.0001 and p=0.005, respectively), which may help explain the etiopathogenesis. However, further data based on large samples and geographically diverse regions are needed (113).

### Others

The detected high level of soluble triggering receptor expressed on myeloid cells-1 (sTREM-1) is dedicated to IGM according to a case-control study. In particular, blockade of TREM may be a promising treatment option for resistant or multiple recurrent patients with IGM (114). In contrast to breast cancer, some preoperative parameters (C-reactive protein, albumin, fibrinogen, fibrinogen/albumin, NLR, etc.) have considerable potential to be early sensitive biomarkers of IGM, providing a useful guide for differential diagnosis of two diseases (115).

## Immunotherapeutic approaches in IGM

### The watch-and-wait approach

Often, no specific treatment is required for IGM, especially for patients with small (<5 cm), unilateral lesions without sinus formation or abscess. IGM is usually a self-limiting inflammatory disease and complete resolution may take 5 to 20 months (116, 117). Observation, known as the watch-and-wait procedure or expectant treatment, seems one of the most appropriate treatment approaches for patients who do no desire medical intervention and are concerned about surgical complications.

In a study of 120 women with IGM, 112 patients recovered spontaneously without surgical or medical treatment (117). A meta-analysis of 10 retrospective studies from five countries unmasked comparable recurrence rates between patients managed with conservative (observation and medicine) and surgical treatment approaches (OR 1.25, 95% CI 0.51 – 3.03) (118). However, a recent meta-analysis of 71 studies including 4735 patients demonstrated a significant difference in relapse rates with expectant management compared with other treatments (p = 0.023) (119). Clinically, treatment plans should be tailored according to symptom severity and patient preference.

### Conventional immunosuppressive agents

#### Steroids

Steroids, also known as corticosteroids or glucocorticoids, are effective and commonly used conservative therapies for IGM patients. Steroids and methotrexate (MTX), with or without surgery, are regarded as the treatment for IGM patients (61). Painful unilateral small (< 5 cm) lesions with minimal discharge or ulceration can be treated with prednisone at a dose of 0.5 mg/kg per day, while those with multiple, bilateral lesions (≥ 5 cm) or significant skin ulcers, secretions/fistulas can be treated with prednisone at a dose of 0.5-1 mg/kg per day or 10-15 mg oral MTX weekly accompanied by daily folic acid supplementation (120, 121). A prospective cohort study of 49 women with biopsy-proven granulomatous mastitis was conducted from 2006 to 2010. Fifty-nine percent of patients initially receiving antibiotics reached incomplete resolution and 90% were prescribed oral steroids. Of those who received steroids, 80% had complete resolution with a median time of 159 days (interquartile range, IQR 120-241 days) (120). A meta-analysis grouped 106 patients into surgical management, oral steroids, and oral steroids plus surgical management groups and found that oral steroids may be a widely acceptable option for patients with concerns about surgical scarring (121).

High-dose prednisolone has a high success rate and a low recurrence rate with a reduction of the need for surgery in treatment of IGM (122). For localized disease, observation, steroids and excision can be applied (123). Under certain circumstances, pregnancy precluded the use of most drugs, but local corticosteroids was effective in terms of treatment response and duration, need for surgery, reduced recurrence and side effects (124). It is generally believed that noninvasive treatments should be initially performed due to the self-limiting nature of IGM. A greater number of valuable studies are needed to determine the appropriate dose of topical steroids and to estimate the efficacy of this therapy (125). The route of administration also influences the efficacy and safety of steroids. A prospective single-arm study reported a novel treatment for patients with nonlactating mastitis (NLM) (27 patients with IGM). After local anesthesia, an appropriate amount of irrigation solution was pumped into the ducts, followed by a daily breast massage lasting for two weeks. During this study, oral antibiotics and fine-needle aspiration for breast abscess were allowed; however, oral intake of corticosteroids and surgical drainage were not allowed. This study found that the 1-year clinical complete response (cCR) rate of NLM patients was more than 90% with insignificant adverse events (126, 127). Based on the available evidence, well-designed randomized controlled trials are encouraged to compare the efficacy and safety of ductal lavage with traditional strategies (surgery, steroids, observation alone, etc.).

Steroids are remarkably useful in acute phase of inflammatory diseases and autoimmune diseases by inhibiting a wide range of immune responses (128). Two decades ago, a basic experiment illustrated that the stability of messenger RNA (mRNA) encoding interleukins, tumor necrosis factor (TNF), and granulocyte-macrophage colony-stimulating factor diminished for the presence of glucocorticoids (129). In the low to moderate dose range, glucocorticoids have variable effects on T lymphocyte subsets. Following glucocorticoid administration, total T cells were slightly reduced in the circulation, while immature CD4+ cells were more affected than mature CD4+ cells, Th17+ cells, and CD8+ cells. All above mentioned subsets transiently increased after hydrocortisone administration (130, 131). The percentage of circulating regulatory T cells increased in patients with lupus or sarcoidosis treated with intravenous methylprednisolone or prednisone (130–132). High dose of glucocorticoids causes a rapid depletion of most circulating T cells due to a combination of effects (e.g. enhanced circulatory emigration (133); inhibition of IL-2 signaling (134); induction of apoptosis) (135–137).

#### Immune modulators

Immunosuppressive drugs are essential in the management of IGM. For patients who failed treatment of antibiotics, steroids and surgery, 94% of them improved and 75% achieved remission after 15 months of treatment with MTX (138). Complete drug-free remission was obtained in 45 (88.2%) of 51 patients treated with immunosuppressive drugs. MTX is preferred as an effective corticosteroid-tapering agent in initial treatment, and azathioprine (AZA) acts as an alternative in patients intolerant to MTX, both of which regulate the inflammatory process and prevent further complications (123, 139–141). Although the treatment algorithm after diagnosis of IGM is still controversial, steroid treatment should be administered first in widespread disease and then MTX or surgery should be considered depending on the response to steroids (123). If IGM patients do not respond to steroids alone, low-dose MTX and prednisone should be considered as an alternative to surgery (142). Meanwhile, the combination of steroids and methotrexate is an effective and reliable option to ensure long-term remission (143). Colchicine and hydroxychloroquine were used in six and four patients with IGM, respectively. Of the six patients treated with colchicine, four did not relapse; and only one patient treated with hydroxychloroquine as a monotherapy healed (11). In 2017, a case of IGM that was refractory to MTX and intralesional and systemic steroids but responded well to mycophenolate mofetil 1500mg twice daily (144), and a recent prospective study also showed the promising results of mycophenolate mofetil in the treatment management of IGM (145).

### Rationale of antibodies and other immune approaches

Several TNF inhibitors were approved by the Food and Drug Administration (FDA). To reduce side effects and costs, most patients with mild or moderate disease are treated with MTX before adding or switching to a TNF inhibitor. These agents may be used alone or in combination with other medications (e.g. prednisone, MTX, hydroxychloroquine). A 32‐year‐old female patient in a pilot report of IGM was successfully treated with etanercept in a combination with MTX; therefore, the improvement achieved with TNF inhibitors in this case offered a different therapeutic option for patients with a similar condition (146). A premenopausal Hispanic woman with refractory IGM had an excellent response to adalimumab despite aggressive steroid and MTX therapy (147). The latest case with overlapping clinical features of IGM, hidradenitis suppurativa, and pyoderma gangrenosum was successfully treated with adalimumab and eventually achieved complete resolution of all lesions without relapse even after discontinuation of biologic therapy (148).

Biologic agents that deplete B cells (e.g., rituximab) and inhibit factors which activate B cells (e.g., belimumab) are both used to treat a variety of rheumatic diseases. In addition to antibody production, B cells present antigens to T cells, activate T cells, and promote the production of proinflammatory cytokines (149). Rituximab, for example, eliminates CD20-positive B cells, induces complement-mediated cytotoxicity, and stimulates apoptosis but has little or nonspecific effects on autoantibody titers; thus, the combination of the above effects may explain disease activity (150).

Several pathways mediating receptor signal transduction have been targeted by orally administered chemical compounds (e.g. small-molecule kinase inhibitors) (151, 152). A pan-janus kinase (JAK) inhibitor was approved in Japan and Korea for the treatment of rheumatoid arthritis patients with an inadequate response to disease-modifying antirheumatic drugs (153, 154); while another selective JAK-1 inhibitor, approved by the European Medicines Agency, showed rapid and significant improvements in disease activity compared with placebo (155, 156). A systematic literature review and meta-analysis of IGM demonstrated the superiority of TNF-blocking agents in combination with MTX (157). A recombinant IL-1 receptor antagonist is significantly less potent than TNF inhibitors in most patients with RA (158, 159). Moreover, the regimen of a IL-1receptor antagonist plus a TNF inhibitor or other biologic agents has not been recommended due to an increased incidence of serious adverse events, including serious infections (160, 161). IL-6 inhibitors have been successfully used in the treatment of RA and other rheumatic diseases (162).

### Characteristics that cannot be explained by immune pathogenesis/unsolved questions and unmet medical needs

#### ANA

Clinical research has not demonstrated the clinical utility of many autoantibodies (e.g. ANA) in IGM (163). Another study did not unravel the autoimmune property by determining the levels of ANA and extractable nuclear antigen (ENA) in patients with IGM (24). Altintoprak and colleagues also evaluated the levels of ANA and ENA in 26 parous patients with IGM, although no indicator for ANA or ENA levels favoring the preeminent impact of autoimmunity on the pathogenesis was found (62).

## Conclusion

IGM is a chronic benign inflammatory breast disease of unknown etiology, but of autoimmune-favorable features such as erythema nodosum and arthritis, coexistence with other autoimmune diseases and seasonal fluctuations in disease onset. Triggering events and precipitating factors are also discussed in this systemic review (including pregnancy and parturition, hormonal disorders, trauma and ductal perforation, smoking, microorganisms, and others). Cellular dysregulation, IL family/cytokines, HLA and others might participate in the immune pathogenesis of IGM. As conventional immunosuppressive agents, steroids and immunomodulators (MTX, AZA, colchicine and hydroxychloroquine) play an important role in treatment. Emerging novel immunological approaches are potential alternatives with a range of biologic agents targeting B cells, kinase inhibitors, and biologic cytokine inhibitors. However, the findings of most clinical studies on autoantibodies do not reach clinical utility and cannot be explained by immune pathogenesis. In the future, more research is expected to focus on the etiology, immunologic mechanism and treatment of IGM.

## Search strategy

PubMed, Scopus, Embase and Cochrane databases were systematically searched for related articles and reviews. The predefined search terms are “IGM,” “idiopathic granulomatous mastitis,” “GLM,” “granulomatous lobular mastitis,” “NPM,” “non-puerperal mastitis,” “NLM,” “nonlactational mastitis,”. Synonyms were combined by the Boolean operator (OR) and paratactic terms were combined by the Boolean operator (AND) when searching relevant content for the corresponding section.

## Author contributions

JY: Conceptualization, Funding acquisition, Methodology, Writing – original draft, Writing – review & editing. XW: Data curation, Formal analysis, Validation, Visualization, Writing – original draft, Writing – review & editing. XH: Conceptualization, Supervision, Writing – review & editing. JZL: Data curation, Formal analysis, Investigation, Visualization, Writing – review & editing. HZ: Conceptualization, Formal analysis, Investigation, Supervision, Validation, Writing – review & editing. HW: Conceptualization, Investigation, Methodology, Supervision, Validation, Writing – review & editing. JL: Conceptualization, Investigation, Methodology, Supervision, Writing – review & editing.
